# The Effect of Carotid Atherosclerosis on the Risk for Open-Angle Glaucoma

**DOI:** 10.1167/iovs.66.9.9

**Published:** 2025-07-02

**Authors:** Victor A. de Vries, Karin A. van Garderen, Caroline C. W. Klaver, Daniel Bos, Maryam Kavousi, Wishal D. Ramdas

**Affiliations:** 1 Department of Ophthalmology, Erasmus Medical Center, Rotterdam, The Netherlands; 2 Department of Epidemiology, Erasmus Medical Center, Rotterdam, The Netherlands; 3 Ophthalmology, Radboud University Medical Center, Nijmegen, The Netherlands; 4 Institute of Molecular and Clinical Ophthalmology, University of Basel, Basel, Switzerland; 5 Department of Radiology and Nuclear Medicine, Erasmus Medical Center, Rotterdam, The Netherlands

**Keywords:** open-angle glaucoma (OAG), carotid artery disease, intraocular pressure (IOP), retinal nerve fiber layer (RNFL)

## Abstract

**Purpose:**

There is evidence that patients with open-angle glaucoma (OAG) have altered hemodynamic parameters of the carotid and retinal arteries. We investigated the effect of carotid artery atherosclerosis on incident OAG, intraocular pressure (IOP), and macular retinal nerve fiber layer (mRNFL) in the general population.

**Methods:**

We analyzed data from the Rotterdam Study, a large prospective population-based cohort study. Carotid atherosclerosis was assessed by measuring the carotid intima-media thickness (cIMT). We used multivariable Cox proportional hazard models to estimate the effect of cIMT on OAG risk, and linear mixed models for IOP and mRNFL. Linear and logistic regressions were used to analyze within participants with OAG the effect of cIMT on age at diagnosis and the requirement for glaucoma surgery.

**Results:**

Among 9652 participants, 193 developed OAG during 89,665 person-years of follow-up (mean follow-up = 10.5 years). Each standard deviation increase in cIMT was associated with a 17% higher risk of developing OAG (adjusted hazard ratio = 1.17, 95% confidence interval [CI] = 1.00 to 1.36, *P* = 0.047). Although no association between cIMT and IOP was found, the mRNFL thickness decreased by an average of 0.17 µm (95% CI = 0.01 to 0.34) for each standard deviation increase in cIMT. Among participants with OAG, an increased cIMT was also associated with a younger age at diagnosis (beta = −1.01 years, 95% CI = −1.84 to −0.17), and more frequent requirement of glaucoma surgery (odds ratio [OR] = 1.93, 95% CI = 1.07 to 3.50).

**Conclusions:**

Among participants with OAG, an increased cIMT was associated with a younger age at diagnosis. In patients with OAG and unexplained continuous progression, a screening ultrasound examination of the carotid arteries might be considered.

Glaucoma is a common neurodegenerative eye disease and the leading cause of irreversible blindness globally.^1^ Among the various forms of glaucoma, open-angle glaucoma (OAG) is the most prevalent, affecting approximately 1% to 3% of middle-aged to elderly individuals.^2^^,^^3^ As the global life expectancy increases, the prevalence of glaucoma is estimated to double over the next 2 decades.^4^

Various etiological pathways underlying OAG have been suggested. There is robust evidence for a mechanical pathway involving mechanical changes at the lamina cribrosa, primarily mediated through elevated intraocular pressure (IOP).^5^^,^^6^ However, OAG can occur in patients with normal IOP (normal tension glaucoma [NTG]), and progression regularly occurs in patients with OAG despite (seemingly) adequate IOP reduction from treatment.^7^^–^^9^ An increasing amount of evidence therefore suggests that OAG is a multifactorial disease with a wider spread of risk factors, several of which present on a continuum and are inter-related. Both hyper- and hypotension have been associated with OAG, as well as type 2 diabetes.^10^^,^^11^ Several genetic variants have been associated with OAG, some of which appear to increase OAG-risk independently of IOP.^12^^,^^13^

There is also evidence linking ocular blood flow to OAG. Some studies have observed that patients with OAG have lower peak systolic velocity, lower end-diastolic velocity, and a higher resistive index in both the carotid and central retinal arteries compared with individuals without OAG.^14^^,^^15^ Reduced ocular blood flow has additionally been associated with OAG in several studies.^14^^,^^16^^,^^17^

However, there are several methodological challenges when measuring retrobulbar blood flow.^18^ Although some factors, such as patient posture, can significantly impact ocular blood flow measurements, a universal standard in methodology in recording measurements has not yet been established.^19^^,^^20^ In addition, the reliability of these measurements depends on the experience of the examiner. Nonetheless, expert training and execution can provide highly reproducible results.^21^^–^^23^

There are also anatomic considerations. The optic nerve head is vascularized through a combination of the central retinal artery, posterior ciliary arteries, choroid, and inner retinal vascular beds, but the relative contribution of these different sources remains incompletely delineated.^24^ Elevated IOP can also result in reduced blood flow to the optic nerve head, further complicating causal inference.^25^ Nevertheless, from an anatomic perspective, the carotid artery is of particular interest because it is the origin of ocular blood flow through the ophthalmic artery and its branches. Atherosclerotic disease in the form of stenotic plaques is common in the carotid arteries. These stenotic plaques are known to substantially affect hemodynamic parameters, limiting post-stenosis blood flow.^26^ Consequently, carotid artery atherosclerosis increases the risk for several adverse neurological outcomes beyond the well-known effect on stroke, such as dementia (including non-vascular dementia) and reduced overall cognitive performance.^27^^,^^28^ It seems therefore likely that carotid atherosclerosis also affects blood flow to the optic nerve, and could increase the risk for OAG. Carotid atherosclerosis is treatable, although not without risks. Understanding the relationship between carotid atherosclerosis and OAG is crucial to be able to make a well-informed treatment decision in patients with OAG with diagnosed carotid artery stenosis, and to gain a deeper understanding of the pathophysiologic mechanisms underlying OAG.

OAG has a substantial hereditary component, with individuals having up to a nine-fold increased risk if they have a first-degree relative with the disease.^29^ For individuals with high genetic risk, any additional provoking factors could be particularly relevant.

Several studies describe changes in ocular perfusion in OAG. In many epidemiological studies, ocular perfusion pressure was calculated using the formula described by Bill et al.^30^^,^^31^ However, we have shown previously that in the Rotterdam Study the association between ocular perfusion pressure with OAG was primarily driven by the inclusion of IOP in the formula to calculate ocular perfusion pressure, not due to an independent effect.^32^ Additionally few to no studies have examined the carotid artery, neither the carotid intima-media thickness (cIMT) nor plaque burden, in relation to OAG in a population-based setting. The few studies that have reported on this relation suffered from small sample sizes, limited data on confounding factors, or misclassification bias due to the use of health-registry data.^33^^–^^35^ Consequently, the relation between the carotid arteries and OAG remains unclear.

We therefore assessed the effect of cIMT, a well-established proxy for carotid atherosclerosis in population-based studies, on OAG in a large prospective population-based cohort.^36^ Additionally, we investigated the association of cIMT with IOP and macular retinal nerve fiber layer thickness (mRNFL). Finally, we analyzed the effect of cIMT on OAG risk specifically in patients whom have a high genetic OAG susceptibility.

## Methods

### Ethics Statement

The Rotterdam Study (RS) has been approved by the Medical Ethics Committee of Erasmus Medical Center (registration number MEC 02.1015) and the Dutch Ministry of Health, Welfare, and Sport (Population Screening Act WBO, license number 1071272-159521-PG). The RS is entered into the Dutch National Trial Register (NTR; www.onderzoekmetmensen.nl) and the World Health Organization (WHO) International Clinical Trials Registry Platform (ICTRP; www.who.int/clinical-trials-registry-plotform) under shared catalog number NTR6831. All participants provided written informed consent following the Declaration of Helsinki to participate in the study and to have their information obtained from their treating physicians.

### Study Population

The RS is a prospective population-based cohort study of people living in Ommoord, a district of Rotterdam, The Netherlands.^37^ The RS consists of four cohorts. The first cohort (RS-I) started in 1991 and consisted of 7983 participants >55 years old (range = 55.0–99.2 years, response rate = 78%). The second cohort (RS-II) started recruiting in 2000 and consisted of 3011 participants >55 years old (range = 55.2–98.9 years, response rate = 67.3%). The third cohort (RS-III) included people aged >45 years old (range = 45.7–90.1 years) and consisted of 3932 participants (response rate = 64.9%) starting from the year 2006. In 2016, recruitment for the fourth cohort (RS-IV) started, targeting participants aged 40 years and older (range = 41.0–96.0 years) and included 3005 (response rate = 45%) participants. Carotid and OAG data were available for a total of 9459 participants, from the first visit of the first 3 cohorts (RS-I-1, RS-II-1, and RS-III-1). Follow-up OAG data was available for all follow-up visits. Follow-up examinations were performed every 4 to 5 years.^37^ Participants underwent an extensive physical examination at the research center as well as a home interview including questionnaires on lifestyle factors and medication usage.

### Ophthalmic Assessment

Participants were examined for the presence of OAG at baseline and were invited for each follow-up round to be re-examined for the presence of OAG. All participants underwent visual field testing. Visual field loss had to be reproducible on 2 screening visual field tests (HFA II 740 or FDT 710; Carl Zeiss, Oberkochen, Germany) and a subsequent HFA 24-2 full threshold or SITA standard test. If no other cause could be identified during the ophthalmic assessment, and no homonymous defects or recognizable patterns like rim artifacts were observed, the defect was considered a glaucomatous visual field loss. Further details were published previously.^2^^,^^3^ We defined OAG as glaucomatous visual field loss in at least one eye, independent of IOP. The IOP was measured using Goldmann applanation tonometry (Haag-Streit AG, Bern, Switzerland).^38^ Optical coherence tomography (OCT) scans were obtained initially using the Topcon 3D-OCT 1000 mk1 and mk2, since August 2011 the Topcon 3D-OCT 2000, and finally since January 2022 the Topcon DRI Triton OCT (Topcon, Tokyo, Japan). Using an in-house deep learning model, based on nnUNetv2 and trained using manually annotated data of 180 participants (708 OCT b-scans),^39^ we calculated the average thickness of the mRNFL over the surface area of the Early Treatment Diabetic Retinopathy Study (ETDRS) grid, excluding the fovea.

### Assessment of Carotid IMT

The carotid arteries were assessed by measuring the cIMT. The cIMT is a well-established predictor of cardiovascular events in population-based studies,^40^ and used as an efficacy endpoint for randomized clinical trials aiming to reduce cardiovascular disease risk.^41^^,^^42^ Acquisition details have been published previously.^40^ In short, measurement of cIMT was performed with ultrasonography of both the left and right carotid arteries using a 7.5 megahertz (MHz) linear array transducer with a Duplex scanner (UltraMark IV; Advanced Technology Laboratories, Bethel, WA, USA). We calculated the cIMT as the average of the mean from the near and far walls measurements of both the right and left carotid arteries.

### Genotyping and Imputation

To identify participants with a high genetic OAG susceptibility, we calculated a genetic risk score (GRS) using genome-wide association study (GWAS) array data and the genome-wide significant variants with beta's from the latest meta-GWAS on OAG.^12^ Genotyping was performed using both the Infinium II HumanHap550(-Duo) (RS-I & RS-II) and 610-Quad Genotyping BeadChip (RS-I & RS-III; Illumina, San Diego, CA, USA) on whole blood samples. Imputation of markers was performed using the TOPMed reference panel.

### Assessment of Covariates

Weight and height were measured at the research center. Body mass index (BMI) was calculated as weight in kilograms divided by the height in meters squared. Blood pressure was measured at the right brachial artery with the participant in a sitting position. Hypertension was defined as a resting blood pressure exceeding 140/90 millimeters of mercury (mm Hg) or the use of blood pressure-lowering medication.^43^ Type 2 diabetes was diagnosed based on the general practitioners’ records, hospital discharge letters, use of blood glucose-lowering medication, or serum glucose measurements (fasting >7.0 mmol/L or nonfasting >11.1 mmol/L).^44^ Hypercholesterolemia was defined as a serum total cholesterol of 239 mg/dL or more, or a high-density lipoprotein cholesterol below 39 mg/dL, or the use of lipid-lowering medication.^45^ History of smoking was assessed during home interviews, and dichotomized as ever having smoked or not. Alcohol consumption (drinks per week) was assessed using food frequency questionnaires during home interviews by trained interviewers.

### Statistical Analyses

Differences in baseline characteristics between those who developed OAG during follow-up and those who did not were analyzed using independent *t*-tests for continuous variables and chi-square tests (or Fisher's exact tests where applicable) for categorical variables. To comprehensively investigate the role of cIMT in OAG, we used the following three-step strategy.

First, we estimated a hazard ratio (HR) with corresponding 95% confidence intervals (CIs) for developing OAG per standard deviation increase in cIMT, using Cox proportional hazard models. If POAG was ascertained at a visit, the event date was assumed to have been at three-fourths between that visit and the prior visit (as the risk would be higher toward the latter half of the interval given the higher risk with increasing age). Participants were censored at death, when lost to follow-up, or at the end of the study period. We estimated a HR for each standard deviation increase in cIMT, and for each quartile of cIMT compared to the first (lowest) quartile. For this analysis, cIMT of the left and right carotid arteries was averaged. Model 1 was adjusted for age, sex, and BMI. We included BMI in model 1 because of its strong correlation with cIMT and because BMI has previously been found to be associated with OAG in the Rotterdam Study.^46^ In model 2, to correct for any potential additional confounding due to cardiovascular comorbidity, we additionally adjusted for hypertension, type 2 diabetes mellitus, and hypercholesterolemia. In model 3, to adjust for any potential confounding due to differences in lifestyle, we additionally adjusted for alcohol intake and smoking. Finally, model 4 incorporated model 3 with the addition of IOP (at baseline), to investigate the IOP-independence. For OAG cases, we used the IOP of the affected eye. If both eyes were affected or unaffected, a random eye was selected. As secondary analysis, we classified all participants with incident OAG with a maximum IOP during follow-up ≤21 mm Hg at any time point (before incidence), as NTG, and the remaining incident OAG cases as high-tension glaucoma (HTG).

Second, we estimated the association between cIMT of each carotid artery (i.e. left and right) at baseline with the ipsilateral IOP and mRNFL thickness (i.e. left carotid cIMT with the mRNFL of the left eye) at the end of follow-up using linear mixed models, with the left and right laterality as random effects with random intercepts and assuming an unstructured covariance matrix. All analyses mRNFL were additionally adjusted for OCT acquisition device. For participants who initiated IOP lowering treatment during follow-up, the last IOP measurement before the treatment was used.

Third, in a case-only analysis, we evaluated the association between cIMT and various glaucoma-related traits (age at diagnosis of OAG, maximum IOP, mRNFL at diagnosis, and requirement of glaucoma surgery). As the number of OAG cases was relatively low, we performed these analyses cross-sectionally using linear and logistic regression models, additionally adjusted for follow-up time to include both prevalent and incident cases. Finally, we stratified the OAG GRS in quintiles, and estimated a HR for cIMT on OAG within each quintile. Because the number of incident OAG cases in quintiles 1 and 2 was prohibitively small (8 and 18 events, respectively), we combined these 2 quintiles as the reference group for this analysis. We also estimated the association of mRNFL with cIMT within each quartile of the OAG GRS. All significance tests were 2-sided, and a *P* value < 0.05 was considered statistically significant. Analyses were conducted with R software version 4.0.3 and R software version 4.2.3 (R Foundation for Statistical Computing), and SPSS version 28.0.1.0 for Windows (IBM Inc., Chicago, IL, USA).

## Results

### Baseline Characteristics

In total, 291 OAG cases and 9459 non-OAG participants were included in this study. Of the 291 OAG cases, 98 were prevalent at baseline and were therefore excluded from the survival analyses. The remaining 193 incident cases were detected during 89,665 person-years of follow-up (mean follow-up = 10.5 years, interquartile range = 5.7, 14.9 years); of these 104 (54%) were classified as NTG cases, and 89 (46%) as HTG cases (Table 1). Compared with participants without OAG, participants with OAG had higher IOP (*P* < 0.001), thinner mRNFL (*P* < 0.001), and a higher OAG genetic risk score (*P* < 0.001; see Table 1). Although over half of all participants had hypertension (55.1%) and a substantial majority (72.7%) were either current smokers or past-smokers, there were no significant differences between participants with and without OAG. Participants with OAG did, however, have a higher prevalence of hypercholesterolemia (see Table 1).

**Table 1.** Baseline Characteristics Presented as Mean ± Standard Deviation at Enrollment, Unless Stated Otherwise

	Participants Without OAG (*N* = 9459)	Participants With OAG (*N* = 291, Including 193 Incidents)	*P* Value
Age, y	62.1 ± 7.9	66.5 ± 7.7	<0.001
Age at diagnosis, y	N/A	74.3 ± 8.8	N/A
Sex, female (*n*, %)	5401 (57.1)	141 (48.5)	0.487
IOP, mm Hg	14.1 ± 3.6	19.3 ± 7.4	<0.001
mRNFL thickness, µm	32.8 ± 5.7	26.6 ± 7.0	<0.001
Glaucoma surgery (*n*, %)	N/A	19 (6.5)	N/A
cIMT, mm	0.8 ± 0.1	0.8 ± 0.2	0.017
BMI, kg/m^2^	27.1 ± 4.1	26.1 ± 3.3	<0.001
Type 2 diabetes (*n*, %)	1653 (17.5)	56 (19.2)	0.487
Hypertension (*n*, %)	5203 (55.0)	166 (57.0)	0.591
Hypercholesterolemia (*n*, %)	5928 (62.7)	204 (70.1)	0.010
Alcohol intake, cups/day; median, IQR	0.6 (0.1, 1.5)	0.5 (0.0, 1.5)	0.445
Smoking, ever (*n*, %)	6876 (72.7)	217 (74.6)	0.721
Standardized OAG genetic risk score^*^	−0.02 ± 0.99	0.84 ± 1.05	<0.001

BMI, body-mass index; cIMT, carotid intima-media thickness; IOP, intraocular pressure; IQR, interquartile range; OAG, open-angle glaucoma; mRNFL, macular retinal nerve fiber layer; N/A, not applicable.

The *P* value refers to independent *t*-tests for continuous variables and chi-square tests for categorical variables.

* Genetic data was available for 8468 (89.5%) controls and 271 (93.1%) cases.

### Intima-Media Thickness and OAG

**Figure 1.**
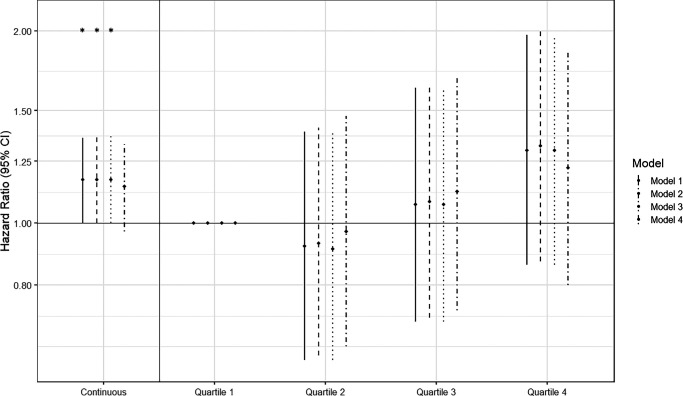
Multivariable adjusted hazard ratios of OAG per standard deviation increase in cIMT (i.e. continuous), and for each quartile of cIMT compared to the lowest quartile, with corresponding 95% confidence intervals. Model 1: Adjusted for age, sex, and body-mass index. Model 2: Model 1 + type 2 diabetes, hypertension, and hypercholesterolemia. Model 3: Model 2 + alcohol intake and history of smoking. Model 4: Model 3 + intraocular pressure. *P < 0.05. cIMT, carotid intima-media thickness; OAG, open-angle glaucoma; 95% CI, 95% confidence interval.

**Figure 2.**
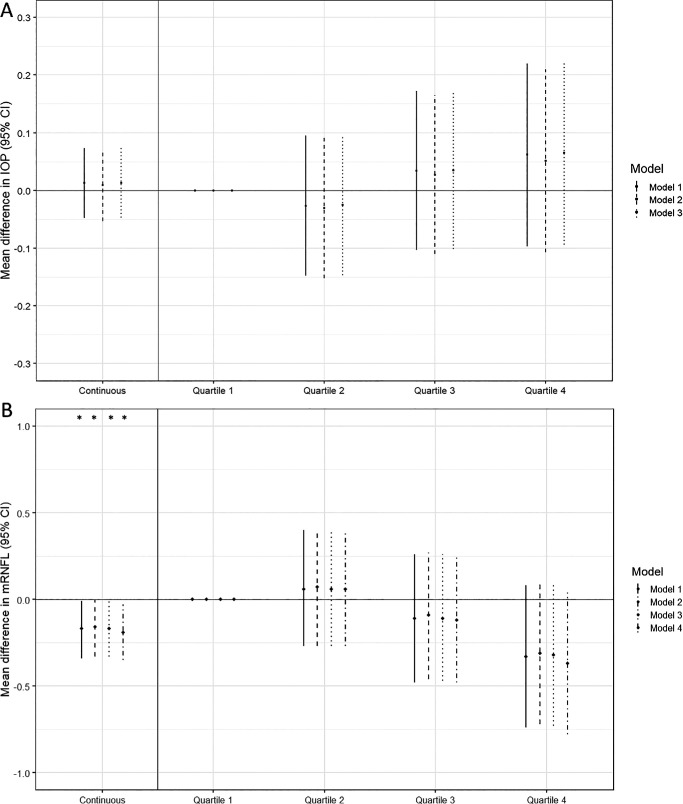
Linear mixed models of IOP (**A**) and mRNFL thickness (**B**) in the general population at the end of follow-up. Effect estimates expressed per standard deviation increase in cIMT, and for each quartile of cIMT compared to the lowest quartile, with corresponding 95% confidence intervals. Model 1: Adjusted for age, sex, and body-mass index (and OCT acquisition device for mRNFL). Model 2: Model 1 + type 2 diabetes, hypertension, and hypercholesterolemia. Model 3: Model 2 + alcohol intake and history of smoking. Model 4: Model 3 + IOP. *P < 0.05. cIMT, carotid intima-media thickness; IOP, intraocular pressure; mRNFL, macular retinal nerve fiber layer; 95% CI, 95% confidence interval.

A larger cIMT significantly increased the risk for OAG, with a HR (95% CI) of 1.17 (95% CI = 1.00–1.36) per standard deviation increase in cIMT (Fig. 1; *P* = 0.047), equal to 1.11 (95% CI = 1.00–1.23) per 0.1 mm increase in cIMT. This effect remained consistent when adjusting for model 2 and model 3. Correcting the model for IOP did attenuate the effect, leaving a similar, albeit non-significant, HR (95% CI) of 1.14 (95% CI = 0.97–1.33). Stratification of the cIMT distribution showed an approximate dose-response relationship, with the highest HR (95% CI) of 1.30 (95% CI = 0.86–1.97) for the participants in the highest quartile of cIMT, although these results were not statistically significant (see Fig. 1). A HR (95% CI) was moderately higher for NTG cases at 1.15 (95% CI = 0.92–1.43), compared to HTG at 1.08 (95% CI = 0.87–1.35), although neither were statistically significant. There was no statistically significant association between cIMT and IOP (Fig. 2A). The mean difference in mRNFL thickness was −0.17 µm (−0.34 to −0.01) with each standard deviation increase in cIMT (Fig. 2B). Similarly to OAG incidence, we observed a trend of thinner mRNFL layer thickness in higher quartiles of cIMT, but none of these associations were significant. Among participants with OAG, cIMT was associated with a younger age at diagnosis of OAG (beta = −1.01 years, 95% CI = −1.84 to −0.17, *P* = 0.018; see Table 2). This effect was more pronounced and remained statistically significant in NTG cases (beta = −1.30 years, 95% CI = −2.56 to −0.05, *P* = 0.042, *N* = 152), but was smaller and no longer significant in HTG cases (beta = −0.43 years, 95% CI = −1.57 to 0.71, *P* = 0.454, *N* = 139). We observed a trend toward a thinner mRNFL at diagnosis, but this was not statistically significant (see Table 2). Participants with OAG did require glaucoma surgery more often with increasing cIMT, with an OR (95% CI) of 1.93 (95% CI = 1.07– 3.50) per standard deviation increase in cIMT.

**Table 2.** Association of Baseline cIMT With Age at Diagnosis and OAG Severity Measures at Diagnosis, Among Participants With OAG Using Linear and Logistic Regression Models

	Beta/SD (95% CI)	*P* Value
Age at Diagnosis, Years^*^		
Model 1	−1.01 (−1.84 to −0.17)	0.018
Model 2	−0.99 (−1.84 to −0.15)	0.021
Model 3	−0.93 (−1.78 to −0.08)	0.032
Model 4	−0.93 (−1.78 to −0.09)	0.031
Maximum IOP, mm Hg		
Model 1	0.26 (−0.55 to 1.08)	0.523
Model 2	0.20 (−0.68 to 1.09)	0.652
Model 3	0.20 (−0.70 to 1.10)	0.661
mRNFL thickness at diagnosis, µm		
Model 1	−0.92 (−2.50 to 0.67)	0.253
Model 2	−0.92 (−2.57 to 0.73)	0.270
Model 3	−0.90 (−2.57 to 0.78)	0.290
Model 4	−0.90 (−2.58 to 0.79)	0.294

Requirement of glaucoma surgery	Odds ratio/SD (95% CI)	*P* Value
Model 1	1.93 (1.07 to 3.50)	0.030
Model 2	1.84 (1.01 to 3.38)	0.048
Model 3	1.85 (1.01 to 3.41)	0.048
Model 4	1.85 (1.01 to 3.41)	0.048

SD, standard deviation.

Expressed as beta or odds ratio per standard deviation increase in cIMT, with corresponding 95% confidence interval.

Model 1: Adjusted for baseline age, sex, body-mass index, and follow-up time (and OCT acquisition device for mRNFL).

Model 2: Model 1 + type 2 diabetes, hypertension, and hypercholesterolemia.

Model 3: Model 2 + alcohol intake and history of smoking.

Model 4: Model 3 + intraocular pressure.

* Not adjusted for follow-up time.

### Intima-Media Thickness and Genetic OAG Risk

First, we validated the association between the OAG-GRS and OAG, which was highly significant (*P* = 4.8 * 10^−40^), with an OR of 2.44 (95% CI = 2.13–2.78) for each standard deviation increase in OAG-GRS. We therefore considered the GRS to be an effective tool for predicting genetic OAG risk within our population. When stratifying in quintiles of the OAG GRS, we did not observe a pattern in the effect of cIMT on OAG (Table 3). Similar to the non-stratified analyses, the estimated effect of cIMT on NTG was moderately higher in the highest quintile of the OAG GRS with a HR of 1.24 (95% CI = 0.87–1.77), compared to a HR of 1.18 (95% CI = 0.84–1.66) for HTG. We also did not observe a clear pattern in the rate of mRNFL thinning in different groups of OAG genetic risk (Table 4).

**Table 3.** Multivariable Adjusted Hazard Ratios of cIMT and OAG, Within Quintiles of an OAG Genetic Risk Score

	Q1 + Q2 (*N* = 2785, 26 Events)	Q3 (*N* = 1383, 22 Events)	Q4 (*N* = 1415, 38 Events)	Q5 (*N* = 1415, 87 Events)
Model 1	1.21 (0.82 to 1.79)	1.29 (0.84 to 1.96)	1.28 (0.88 to 1.86)	1.23 (0.97 to 1.57)
Model 2	1.22 (0.83 to 1.80)	1.20 (0.77 to 1.88)	1.31 (0.89 to 1.92)	1.25 (0.98 to 1.60)
Model 3	1.25 (0.85 to 1.85)	1.19 (0.76 to 1.85)	1.32 (0.91 to 1.93)	1.25 (0.98 to 1.60)
Model 4	1.27 (0.86 to 1.89)	1.18 (0.74 to 1.87)	1.35 (0.92 to 1.97)	1.29 (1.01 to 1.66)^*^

Q, quintile.

Effect estimates expressed per standard deviation increase in cIMT with corresponding 95% confidence intervals.

Model 1: Adjusted for age, sex, and body-mass index.

Model 2: Model 1 + type 2 diabetes, hypertension, and hypercholesterolemia.

Model 3: Model 2 + alcohol intake and history of smoking.

Model 4: Model 3 + intraocular pressure.

* *P* < 0.05.

**Table 4.** Linear Mixed Models of cIMT and Macular Retinal Nerve Fiber Layer Thickness (at End of Follow-Up), Within Quartiles of an OAG Genetic Risk Score

	Q1 (*N* = 1080 Eyes)	Q2 (*N* = 1076 Eyes)	Q3 (*N* = 1074 Eyes)	Q4 (*N* = 1070 Eyes)
Model 1	−0.14 (−0.47 to 0.19)	−0.33 (−0.63 to −0.02)^*^	0.02 (−0.35 to 0.39)	−0.23 (−0.58 to 0.12)
Model 2	−0.01 (−0.34 to 0.31)	−0.20 (−0.51 to 0.11)	0.10 (−0.27 to 0.46)	−0.20 (−0.54 to 0.14)
Model 3	−0.01 (−0.33 to 0.31)	−0.21 (−0.52 to −0.10)	0.13 (−0.24 to 0.50)	−0.20 (−0.54 to 0.14)
Model 4	−0.18 (−0.34 to 0.30)	−0.22 (−0.53 to 0.09)	0.07 (−0.30 to 0.44)	−0.19 (−0.53 to 0.16)

OCT, optical coherence tomography.

Effect estimates expressed per standard deviation increase in cIMT with corresponding 95% confidence intervals.

Model 1: Adjusted for age, sex, body-mass index, and OCT-device.

Model 2: Model 1 + type 2 diabetes, hypertension, and hypercholesterolemia.

Model 3: Model 2 + alcohol intake and history of smoking.

Model 4: Model 3 + intraocular pressure.

* *P* < 0.05.

## Discussion

### Summary of Findings

In this prospective population-based cohort study, we observed an increased risk to develop OAG, and reduced mRNFL, in participants with a higher cIMT at baseline. Within participants with OAG, higher cIMT was also associated with younger age at diagnosis and more frequent requirement of glaucoma surgery. We found no association between cIMT and IOP. Finally, we observed no consistent pattern in the effect of cIMT on OAG in different strata of genetic OAG risk.

### Relation With Literature

Unfortunately, data on carotid atherosclerosis and OAG is sparse. Previously in 2006, we did not find an association between OAG and cIMT, with an OR (95% CI) of 0.86 (95% CI = 0.47–1.57) for the highest tertile compared to the lowest.^33^ However, the number of OAG cases and follow-up at that time was relatively short: 87 incident cases detected over 7 years of follow-up, versus 193 cases detected over up to 30 years of follow-up in the present study. In 2007, a follow-up study was performed examining arterial stiffness of the carotid wall with OAG by Hulsman et al.^34^ This yielded a more suggestive OR of 2.84 (95% CI = 0.99–8.10) for HTG when comparing the highest tertile to the lowest; however, probably due to a lack of statistical power this result was also not statistically significant. Our current sample size is substantially larger, and perhaps more importantly, we have been able to collect over 30 years of OAG follow-up data for a far more accurate estimate of OAG incident risk. We did find a statistically significant effect with a HR of 1.17 (95% CI = 1.00–1.36), with a much more modest effect estimate compared with previous findings. Since the work of de Voogd and Hulsman et al., the role of carotid atherosclerosis in OAG pathogenesis has remained largely unexamined in the literature. Many studies describe changes in ocular perfusion in OAG, but few to none examined the carotid artery, despite it being the source of that blood flow. One cohort study was published by Chou et al., examining the relation between carotid atherosclerosis and OAG using data from a Taiwanese national health-registry database.^35^ Analyzing 2093 patients with carotid atherosclerosis and 8372 healthy controls matched by age and sex, they found a statistically significant HR of 1.50 (95% CI = 1.11–2.02) for OAG. Unfortunately, the interpretation of health-registry based results is challenging, as the exposure, outcome, and covariate ascertainment has substantial limitations. For example, approximately 50% of all OAG cases in developed nations remain undiagnosed, and will therefore be misclassified as healthy controls in a health registry. Similarly, carotid atherosclerosis is typically asymptomatic and will therefore also be regularly misclassified. Nevertheless, the consistent direction of effect of their results compared to ours, strengthens the hypothesis that carotid atherosclerosis increases the risk for OAG.

Despite both cIMT and OAG being associated with several cardiovascular risk factors, most notably hypertension and BMI, we did not observe any substantial change in any of our analyses when adjusting for cardiovascular comorbidities. Similarly, adjusting for lifestyle factors did not significantly change the observed effects. This suggests that the increased OAG risk with increasing carotid atherosclerosis could be due to a more direct causal effect. Interestingly, some hypoglycemic drugs, most notably exenatide, alogliptin, and metformin, have been shown to reduce cIMT with long-term use.^47^ Previously, we reported that patients with type 2 diabetes mellitus treated with metformin had a substantially lower risk for OAG with an OR (95% CI) of 0.18 (95% CI = 0.08–0.41).^11^ The biological mechanism for this protective effect has yet to become clear. We hypothesize that it might be partially mediated through improved carotid vasculature health.

We observed no consistent pattern in the effect of cIMT on OAG in participants with low or high genetic risk for OAG. To our knowledge, our study is the first to analyze this relationship. Most, although not all, genetic OAG variants have been shown to affect the mechanistic pathway, either through elevation of IOP or by increasing the IOP susceptibility of the optic nerve head.^23^ The lack of interaction between OAG genetic risk and cIMT therefore supports our hypothesis that carotid atherosclerosis affects OAG through a non-mechanistic (i.e. IOP independent) vascular pathway. If cIMT increases OAG risk in an IOP-independent manner, we would expect it to explain a larger proportion of the NTG cases compared to HTG. Although these sensitivity analyses were not statistically significant, we observed a moderately larger effect estimate for NTG compared to HTG relative risk, further reinforcing our hypothesis.

### Strengths and Weaknesses

The greatest strength of our study is its population-based design, allowing us to estimate the relation between cIMT and OAG in the general population. Our results are, therefore, broadly generalizable in a European population. Second, cardiovascular measures, such as cIMT, are often strongly correlated with a wide range of lifestyle factors and other cardiovascular conditions. As a result, confounding bias through these shared factors is an ever-present threat. Our comprehensive data allowed us to adjust for a wide range of covariates to alleviate this concern. Interestingly, adjusting for these covariates did not substantially attenuate our results, suggesting that the increased risk is a true causal effect of carotid atherosclerosis on OAG incidence. Third, in our study, we assessed cIMT at baseline in a large cohort and OAG for a long follow-up period of up to 25 years, giving us the statistical power to detect significant effects without compromising study design (i.e. reverting to a retrospective and/or case-control design). Finally, our definition of OAG required the presence of a confirmed visual field defect. Misclassification due to ocular hypertension and/or physiological optic nerve head variation was therefore avoided, and our estimates can be interpreted as the true effects on OAG risk.

Some limitations should be considered. Our population-based design does give an accurate representation of the general population, but due to the relatively low incidence of OAG this design does remain limited by the number of incident OAG cases. Especially in the stratified analyses, our results seem likely to have been more consistently significant with a larger number of events. Similarly, analyzing progression within OAG cases might have been more feasible had the number of OAG cases been higher. Second, we did not perform laterality-specific analyses for OAG risk. The observed HR of 1.17 per standard deviation increase of the average cIMT might underestimate the effect of unilateral vascular changes affecting the corresponding eye. Third, we measured neither the blood flow of the carotid arteries, nor ocular or retrobulbar blood flow. Combining our analyses with these measurements or data from OCT angiography might have allowed for more direct and detailed connections to be drawn from carotid to ocular blood flow and eventually OAG. Fourth, we cannot exclude selection bias due to the selective participation rate among individuals with either carotid atherosclerosis and/or OAG. Participation in population-based studies is generally associated with higher social status, female sex, healthier lifestyle, and prevalence of disease.^48^ In the first subcohort (RS-I) of our study, nonresponders were indeed on average (95% CI) 3.36 years (95% CI = 2.89–3.82) older than responders, although there was no significant difference in sex distribution between nonresponders and responders (proportion of female subjects = 63.1% vs. 61.1%, *P* = 0.08). Our participation rates (65%–78%) are, however, remarkably high for a population-based study. In comparison, the baseline participation rate was only 5.5% for the UK-Biobank, 31.8% for the LIFE-Adult study, and 39% for the EPIC-Norfolk study.^48^^–^^50^ We have therefore minimized the risk of selection bias as much as is reasonably feasible in a population-based setting, although some risk remains. Fifth, cIMT was associated with increased overall mortality in our study (HR = 1.14, 95% CI = 1.11–1.17, *P* < 0.001). Participants with higher cIMT might have died before developing OAG. The actual effect of cIMT on OAG might therefore be less modest than we found in our study. Finally, although cIMT is the most commonly used proxy for carotid atherosclerosis in population-based studies, it is not always reflective of atherosclerotic stenosis. Therefore, although a higher cIMT increases the risk of OAG, this might not be directly 1:1 applicable to a clinical carotid stenosis diagnosis.

### Conclusions and Future Considerations

Our study demonstrates that a higher cIMT increased the risk for OAG independent of IOP. Further prospective cohort studies are needed to confirm this relation and to identify potential subgroups with glaucoma susceptibility due to compromised carotid flow. Additionally, future research should combine carotid atherosclerosis measurements with ocular or retrobulbar blood flow measurements to further elucidate underlying pathomechanisms. In patients with diagnosed carotid artery stenosis, it's potential effect on OAG incidence and/or progression should be considered when making a treatment decision. In patients with OAG and continuous progression despite an (seemingly) adequate IOP reduction, a screening ultrasound examination of the carotid arteries might be considered.
